# NIA Intramural Research Program

**DOI:** 10.1093/geroni/igaf122.1560

**Published:** 2025-12-31

**Authors:** Eleanor Simonsick

**Affiliations:** National Institute on Aging, Baltimore, Maryland, United States

## Abstract

Dr. Simonsick will discuss the work of the NIA Intramural Research Program. Dr. Simonsick will also be available for small group discussion.

